# Assessment of neutralization susceptibility of Omicron subvariants XBB.1.5 and BQ.1.1 against broad-spectrum neutralizing antibodies through epitopes mapping

**DOI:** 10.3389/fmolb.2023.1236617

**Published:** 2023-09-27

**Authors:** Masaud Shah, Hyun Goo Woo

**Affiliations:** ^1^ Department of Physiology, Ajou University School of Medicine, Suwon, Republic of Korea; ^2^ Department of Biomedical Science, Graduate School, Ajou University, Suwon, Republic of Korea

**Keywords:** SARS-CoV-2, neutralization, broad-spectrum, Omicron, BQ.1.1, XBB.1.5, antibodies

## Abstract

The emergence of new variants of the SARS-CoV-2 virus has posed a significant challenge in developing broadly neutralizing antibodies (nAbs) with guaranteed therapeutic potential. Some nAbs, such as Sotrovimab, have exhibited varying levels of efficacy against different variants, while others, such as Bebtelovimab and Bamlanivimab-etesevimab are ineffective against specific variants, including BQ.1.1 and XBB. This highlights the urgent need for developing broadly active monoclonal antibodies (mAbs) providing prophylactic and therapeutic benefits to high-risk patients, especially in the face of the risk of reinfection from new variants. Here, we aimed to investigate the feasibility of redirecting existing mAbs against new variants of SARS-CoV-2, as well as to understand how BQ.1.1 and XBB.1.5 can evade broadly neutralizing mAbs. By mapping epitopes and escape sites, we discovered that the new variants evade multiple mAbs, including FDA-approved Bebtelovimab, which showed resilience against other Omicron variants. Our approach, which included simulations, endpoint free energy calculation, and shape complementarity analysis, revealed the possibility of identifying mAbs that are effective against both BQ.1.1 and XBB.1.5. We identified two broad-spectrum mAbs, R200-1F9 and R207-2F11, as potential candidates with increased binding affinity to XBB.1.5 and BQ.1.1 compared to the reference (Wu01) strain. Additionally, we propose that these mAbs do not interfere with Angiotensin Converting Enzyme 2 (ACE2) and bind to conserved epitopes on the receptor binding domain of Spike that are not-overlapping, potentially providing a solution to neutralize these new variants either independently or as part of a combination (cocktail) treatment.

## Introduction

SARS-CoV-2 neutralizing antibodies (nAbs) have thus far played a crucial role in preventing and treating COVID-19, but they can be hindered by viral evolution and the virus’s ability to evade the host immune response (Cox et al., 2023; Miller et al., 2023). This was particularly demonstrated by the emergence of highly contagious BA.1 sublineage in November 2021 and several other variants of concern (VOCs) since the start of the pandemic (Brown et al., 2022). The evolution of the Omicron has led to the emergence of new subvariants, including BA.2.75.2, BA.4.6, BQ.1.1, and XBB.1.5 (Callaway, 2023), which are highly transmissible and evade the immune system even in vaccinated individuals (Brown et al., 2022; Tamura et al., 2022; Lasrado et al., 2023). Approximately 80% of the population has been infected with at least one of the Omicron subvariants within a year, due to the lack of effective vaccination (Brown et al., 2022; Lin et al., 2023; Zou et al., 2023). Recent studies have shown that the Omicron subvariants are escaping from neutralization induced by current vaccines, raising concerns about their potential to infect individuals who have received three or four vaccine doses, including a bivalent booster (Lin et al., 2023; Miller et al., 2023; Zou et al., 2023). The new subvariants, particularly XBB.1.5 became prevalent in many countries by mid-2023 due to their additional mutations in the spike.

To be ready for future variants and sarbecovirus pandemics, it is necessary to develop broad-spectrum antibody therapeutics and vaccines. However, we still lack a complete understanding of the Spike epitopes that can induce broad sarbecovirus neutralization. In response to the escalation of the COVID-19 pandemic, many initiatives have been launched to find treatments, including studies on existing medications. Sharing information and resources will help explore potential solutions and increase the chances of finding an immediate and lasting treatment.

A recent cohort study has identified a subset of individuals as “elite neutralizers” with broad-spectrum neutralizing antibodies (broad-nAbs) that neutralize SARS-CoV-2 VOCs including Omicron BA.5 (Vanshylla et al., 2022). While some of these monoclonal antibodies could neutralize the subvariants, others escaped due to single-point mutations in the spike (Gruell et al., 2022a). Using our expertise in computational antibody design, we have created models of the broad-nAbs and mapped their conserved epitopes on the receptor binding domain (RBD) of Spike. This comprehensive mapping of conserved sites provides important guidelines for the development of broad-spectrum therapeutics against BQ.1.1, XBB.1.5, and perhaps other emerging variants sharing the mapped epitopes.

## Results

### RBD class designation of the broad-nAbs

**FIGURE 1:**
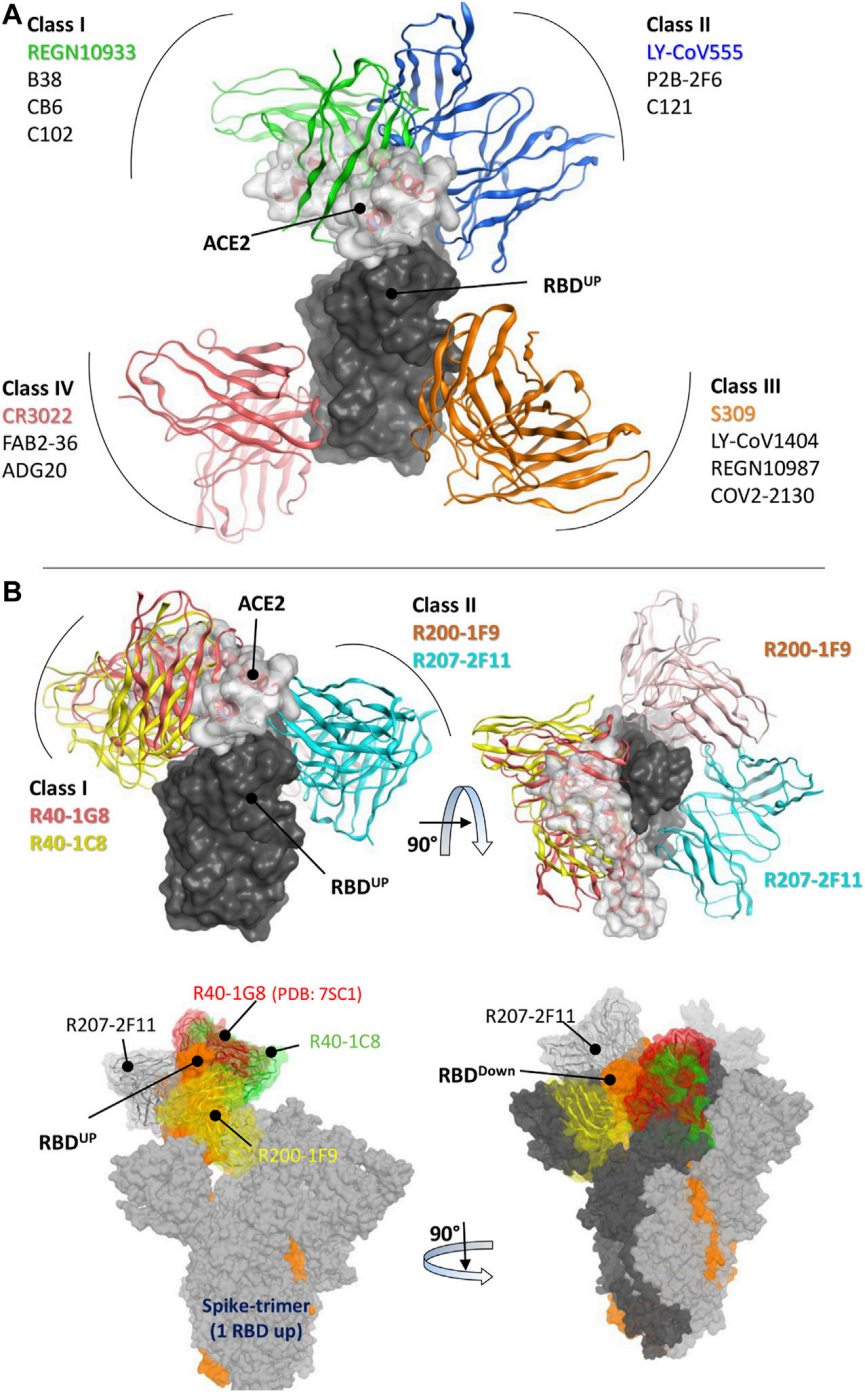
Epitope-based Classification of RBD-Binding Antibodies and Class Designation of Broad-Neutralizing Antibodies (nAbs). **(A)** Class I and Class II Monoclonal Antibodies (mAbs) interfere with the Angiotensin-Converting Enzyme 2 (ACE2) receptor (represented by the transparent surface), while Class III and Class IV mAbs bind outside the ACE2 interface. [**(B)**, top] The mAbs R40-1G8 and R40-1C8 directly compete with the ACE2 receptor, while R200-1F9 and R207-2F11 do not. [**(B)**, bottom] All four mAbs are bound to the trimeric Spike (wild type) protein. Only R207-2F11 accommodates both the up and down conformations, while the other mAbs bind exclusively to the RBD down conformation.

The RBD-binding antibodies are structurally characterized into 4 classes based on their binding epitopes, their ability to bind an ‘‘up’’ or ‘‘down’’ RBD conformation, and interference with Angiotensin Converting Enzyme 2 (ACE2) binding (Vanshylla et al., 2022). Class I antibodies such as C102 block ACE2, bind only to the “up” RBD conformation, and have relatively shorter CDRH3 loops (Barnes et al., 2020a). Class II antibodies bind to both “up” and “down” RBD conformations, interact with adjacent RBDs, and neutralize the Spike-ACE2 interaction (Figure 1A). Class III antibodies bind outside the ACE2-binding site, while Class IV antibodies do not block ACE2 and bind only to the “up” RBD conformation (Barnes et al., 2020a). It has been shown that Class I antibodies with short CDRH3 and class II with long CDRH3 are typically knocked out by Lys417 or Glu484 mutants, respectively (Wu et al., 2020; Yuan et al., 2020). However, the current cohort study has identified several IGHV3-53 antibodies that defy this proposed paradigm (Gruell et al., 2022b). These antibodies, with 93.5%–97.3% germline identity, have demonstrated resistance to the typical variant escape due to minor differences in their antibody sequences (Vanshylla et al., 2022).

Three broad-nAbs R40-1G8, R40-1C8, and R207-2F11 investigated here are encoded by IGHV3-53 but different light V genes (KV1-9, KV1-9, and KV1-33, respectively). Only R200-1F9 is encoded by the IGHV3-48 gene with longer CDRH3 (Table 1). Although R40-1G8 and R207-2F11 share identical CDRH3 and logically they should follow the typical class I rule, both antibodies were found to respond differently to SARS-CoV-2 variants in terms of neutralization. R40-1G8 failed to neutralize all previous Omicron variants while R207-2F11 retained its neutralization capacity (Vanshylla et al., 2022). In addition, our epitope mapping suggests that R40-1C8 overlaps with R40-1G8 and partially competes with ACE2 (Figure 1B), yet the former neutralized all but BA.4/5 variant of Omicron while the latter was devoid of this potential (Gruell et al., 2022b). This notion suggests that while many IGHV3-53 encoded antibodies may agree to the epitope-based classification of RBD-binding mAbs, some defy this rule.

**TABLE 1:** SARS-CoV-2 broad-nAbs and their heavy and light chains encoding genotypes.

Name	Heavy V	CDRH3	Heavy J	Light V	Light J
R200-1F9	IGHV3-48	VRDARDGHSNNDFDY	IGHJ4	IGKV3-11	IGKJ5
R207-2F11	IGHV3-53	ARDLVYRGMDV	IGHJ6	IGKV1-33	IGKJ4
R40-1G8	IGHV3-53	ARDLYVFGMDV	IGHJ6	IGKV1-9	IGKJ2
R40-1C8	IGHV3-53	VRDLVDYGMDV	IGHJ6	IGKV1-9	IGKJ2

After extensive Ag-Ab docking simulations and epitope mapping (discussed below), we designated R40-1G8 and R40-1C8 as class I and R200-1F9 and R207-2F11 as class II antibodies. Both R40-1G8 and R40-1C8 compete with ACE2 while R200-1F9 and R207-2F11 do not (Figure 1B). Subsequently constructing full-length trimeric Spike-mAbs models, we propose that R207-2F11 with shorter CDRH3 can bind RBD in both up and down conformation without making any clash with the adjacent RBD, while R200-1F9 with longer CDRH3 may bind the up conformations only (Figure 1B). In their cryo-EM analysis, Vanshylla et al. (2022) proposed that R40-1G8 Fab bind to both up (state 1) and down (state 2) RBDs; however, due to the relatively low resolution for the RBD and Fab in state 2, they could build a model for state 1 only. Based on our trimeric Spike models and the reported epitope of R40-1G8, we demonstrated that it is not plausible for this mAb to bind the down conformation of RBD, regardless of the up or down conformation of the nearby RBD (discussed in detail below). Based on their non-overlapping epitopes on RBD and the fact that R207-2F11 can potentially bind to both up and down conformation, R40-1C8, R200-1F9, and R207-2F11 could be used as cocktail therapy as they hold potential neutralization against Omicron and other variants of SARS-CoV-2 (Gruell et al., 2022b).

### All Omicron subvariants escape R40-1G8 broad-nAb

**FIGURE 2:**
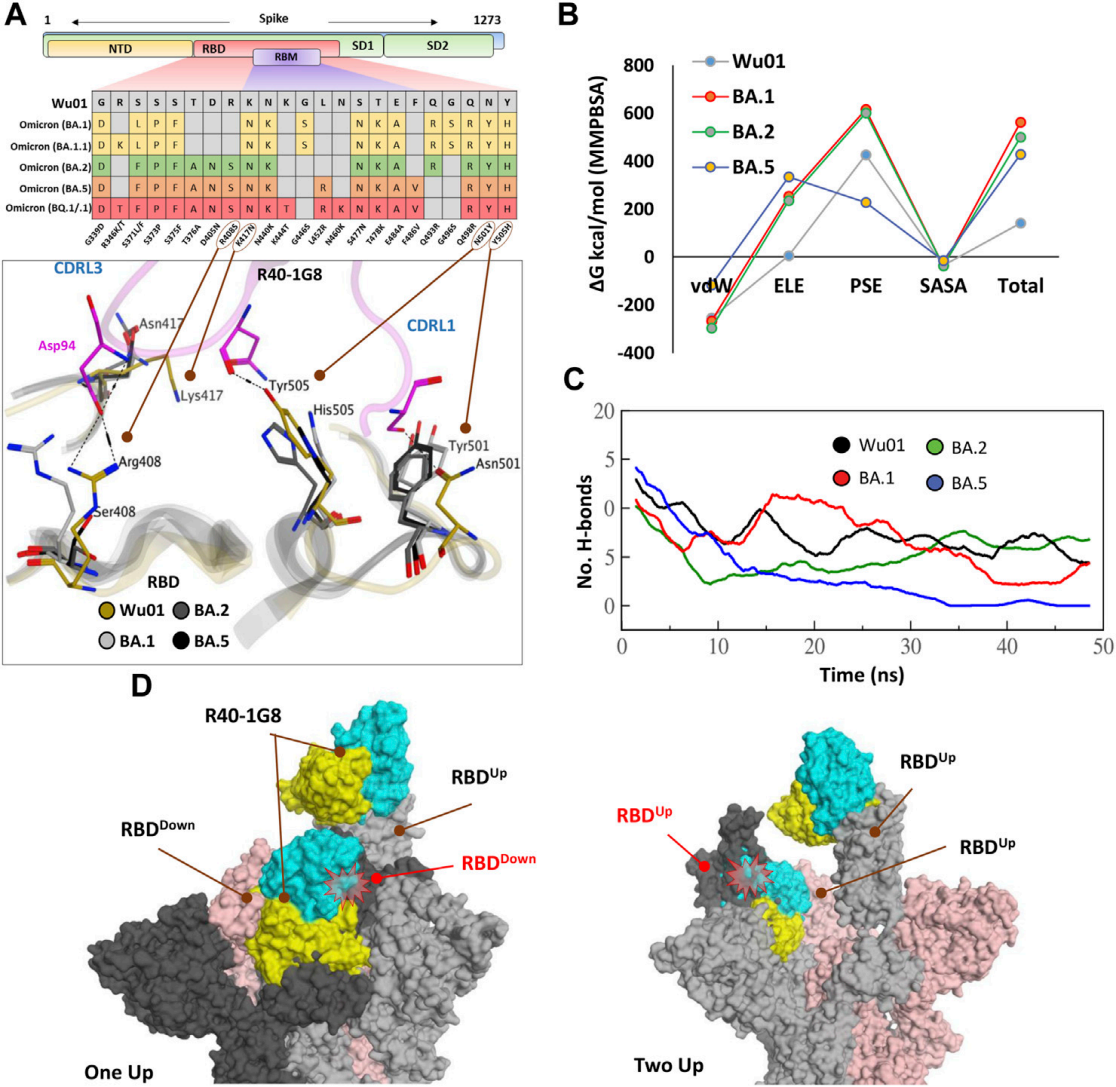
The escape of Omicron subvariants from broad-nAb R40-1G8. **(A)** Mutations in the RBD region of Spike protein of SARS-CoV-2 Omicron variants and their effect on the interface contacts with respect to R40-1G8. Multiple mutations at the interface are indicated with stick and circles **(B)** Changes in the binding free energy (BFE) of R40-1G8 bound to the SARS-CoV-2 variants. **(C)** Changes in the number of Hydrogen bonds between R40-1G8 and respective variants. **(D)** The R40-1G8 Antibody can only be accommodated onto the RBD in its “up” conformation in the trimeric Spike model. The Fab bound to the down conformation of the RBD clashes with the adjacent RBD in both the up and down states.

R40-1G8, one of the broad-nAbs and classified as class I, can potentially neutralize Wu01 strain, several other SARS-CoV-2 variants, and 17 variants with single amino acid mutations at the potential RBD escape sites (Vanshylla et al., 2022). Interestingly, most of these mutations are now reported in the emerging Omicron variants (Figure 2A). However, Florian Klein’s group has recently reported in their pre-print study that all Omicron variants are showing some resistance to R40-1G8 (Gruell et al., 2022b). This suggests that R40-1G8 can potentially endure the interface changes brought by a single mutation; however, multiple mutations in the same epitope may force conformation changes in the RBD that obscure the Ag-Ab interface and CDRs-fitting onto their epitopes.

To expand upon the binding mode and investigate the escape of Omicron subvariants from this broad-nAb, we constructed their protein models and explored their interfaces. As discussed above, R40-1G8 can resist single mutation at Lys417 and other positions; however, a single amino acid variant at Arg408 was not investigated which appeared in the BA.2, BA.4/5, and BQ.1/.1 variants as Arg408Ser (Supplementary Figure S1A). This is the only amino acid that established an electrostatic bond with the CDRL2 Asp94 and apprehended the R40-1G8-RBD complex, which is otherwise lost in Lys417/Asn and Arg408/Ser double mutant in BA.2, BA.4/5, BQ.1/.1, and XBB.1.5 variants (Figure 2A; Supplementary Table S1). The idea that Lys417/Asn single variant did not show resistance to R40-1G8 in the previous findings (Vanshylla et al., 2022) was due to the contribution of the strong electrostatic bond by Arg408 and therefore defied the typical class I antibodies knockout phenomena by Lys417 mutation (Barnes et al., 2020a; Harvey et al., 2021).

We took advantage of the molecular dynamics simulation (MDS) and endpoint binding free energy (BFE) calculation to validate this loss in the neutralization potential of R40-1G8 against Omicron and its subvariants. The average binding free energy of 200 structural representatives sampled from a 50ns trajectory suggests that R40-1G8-RBD complexes of the BA.1, BA.2, and BA.5 subvariants gain significant energy along the course of simulation (Figure 2B; Supplementary Figure S1B). This increase in the total BFEs indicates the destabilization of the Ag-Ab complexes, which could be attributed to the rise in electrostatic energy (ELE); in other words, the rise in ELE is due to the omission of crucial Arg408-Asp94 salt bridge upon Arg408Ser mutation (Figure 2A). Although MMPBSA-based BFE estimation is quite robust (Kumari et al., 2014), we went on to confirm this change in energy by single frame MMGBSA method (Genheden and Ryde, 2015). The total BFE of RBD^Wu01^-nAb was recorded as −110.03 kcal/mol and that of BA.1, BA.2 and BA.5 were −71.97 kcal/mol, −93.66 kcal/mol, and −63.37 kcal/mol, respectively. Only RBD^Wu01^-nAb had ELE as −99.52 kcal/mol while other complexes had this energy-term over 157.03 kcal/mol (Supplementary Table S2). This was further supported by the sudden decline in the hydrogen bonds between nAb and BA.2 and BA.5 RBDs containing Arg408Ser substitution (Figure 2C). To track this complete loss in the hydrogen bonds network between RBD^BA.5^-nAb, we sampled 1,000 frames from the 50 ns MDS trajectory and looked for the RBD-Ab separation as a function of time. The fab and RBD molecules completely separated in RBD^BA.5^-nAb but remained intact in RBD^Wu01^-nAb (Supplementary Video S1).

Generally, Class I RBD antibodies can bind to only up conformation of the RBD (Barnes et al., 2020b; Barnes et al., 2020c); surprisingly, R40-1G8 has been reported to bind both up and down conformation of RBD in a trimeric Spike model. In their explanation, Vanshylla et al. suggest that “in the R40-1G8-spike map, there is one ‘‘up’’ RBD with R40-1G8-Fab, one ‘‘up’’ RBD without R40-1G8-Fab, and a ‘‘down’’ RBD with R40-1G8-Fab” (Vanshylla et al., 2022). To debate this further, we constructed two trimeric Spike models with two R40-1G8 Fabs; 1) containing one RBD^Up^ bound to the Fab and one of the two RBD^Down^ bound to the second Fab, 2) containing two RBD^Up^ with one empty and the other bound to Fab, and the third RBD^Down^ was bound to Fab (Figure 2D). We could demonstrate that R40-1G8 Fab freely binds to the RBD^Up^ conformation; however, following the epitope suggested by Vanshylla et al. the second Fab cannot be accommodated and fit onto the RBD^Down^, irrespective of the down or up conformation of the adjacent RBD. In both scenarios, Fab bound to the down RBD clashes with the adjacent RBD, confirming the general concept of class I RBD antibodies (Figure 2D). Since these models are based on the actual epitopes suggested by the same groups and therefore dependable, we believe the alternative binding pose of R40-1G8 onto the down RBD could be an artifact or a transient low-affinity epitope on RBD which was captured during cryo-EM scanning (Vanshylla et al., 2022). Overall, these data suggest that all Omicron variants escape from an R40-1G8 due to the collective contribution of multiple mutations in the conformational rearrangement of a relatively conserved epitope.

### R40-1C8 neutralize all SARS-CoV-2 variants except BA.4/5 BQ.1.1 and XBB.1.5

**FIGURE 3:**
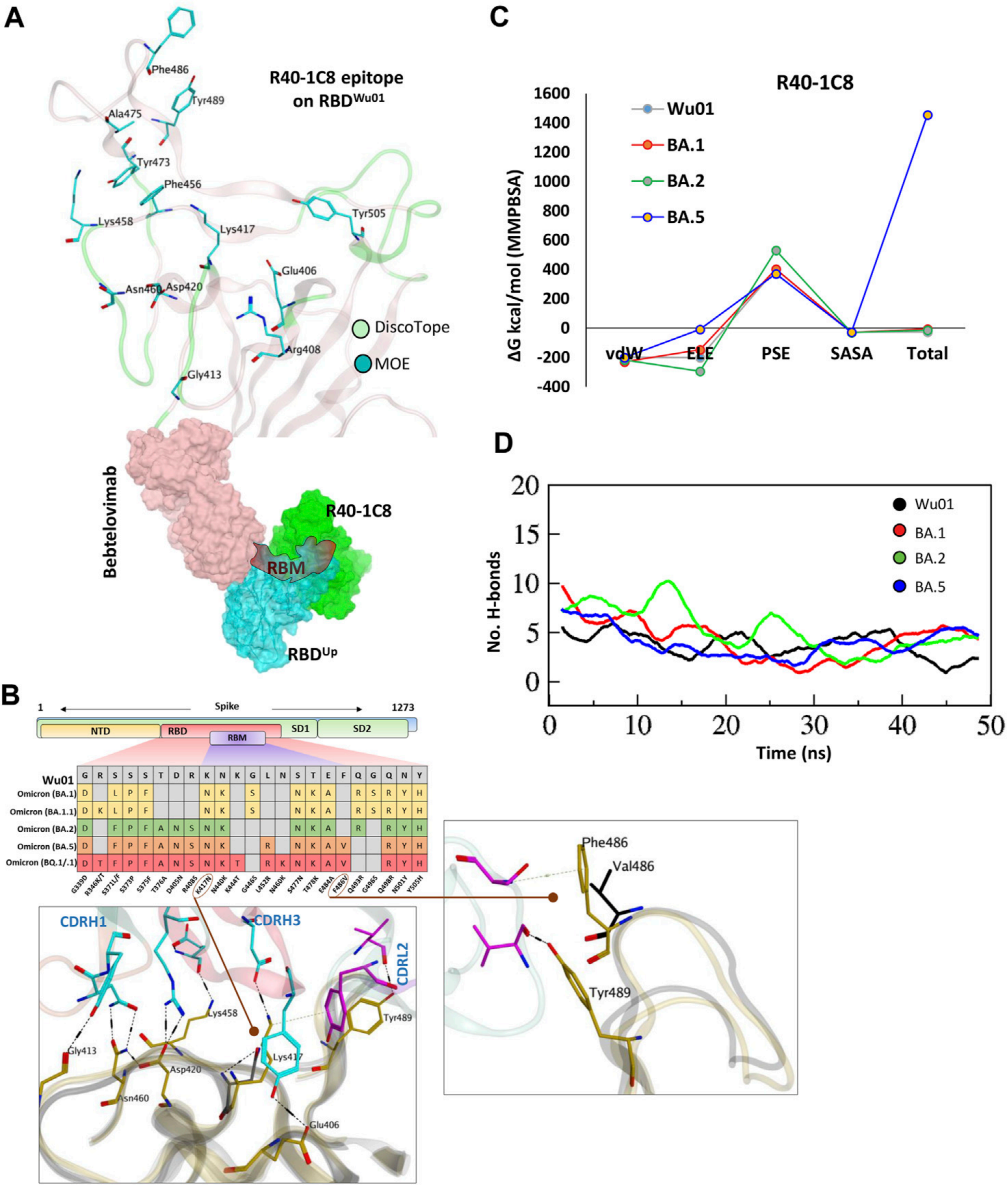
The epitope mapping and escape of BA.5, BQ.1.1, and XBB.1.5 subvariants from R40-1C8. **(A)** The epitope residues on RBD are predicted through DiscoTope (green color cartoon representation) and Molecular Operating Environment (MOE, Cyan color). The surface maps (colored according to chains) shows that R40-1C8 does not compete with Bebtelovimab. **(B)** Mutations in the RBD region of Spike protein of SARS-CoV-2 Omicron variants and their effect on the interface contacts with respect to R40-1C8. Mutations [including Phe(F)486Val(V)] at the interface are indicated with stick and circles. **(C)** Changes in the binding free energy of R40-1C8 bound to the SARS-CoV-2 variants. **(D)** Changes in the number of Hydrogen bonds between R40-1G8 and respective variants.

As discussed in the class designation, R40-1C8 is a class I RBD nAb and potentially binds to its up conformation only, partially competing with R40-1G8 but not Bebtelovimab (an FDA-approved COVID-19 broad-spectrum therapeutic mAb) (Figure 3A). However, neutralization assay suggests that, unlike R40-1G8, R40-1C8 exhibits significant neutralization against all Omicron subvariants (IC50 <0.085 μM/mol) but not BA.4/5 (Gruell et al., 2022b; Vanshylla et al., 2022). Three positively charged residues Arg408, Lys417, and Asp420 can potentially establish salt bridges with the CDRH3; here only the latter two could make such bonds (Figure 3B; Supplementary Table S3). The loss of Lys417 salt bridge by BA.1 and BA.2 but their susceptibility to R40-1C8 neutralization suggest that Asp420-CDRH3 contact compensates for this loss and holds the RBD-Ab complex intact. A somewhat similar effect was previously reported, where R40-1G8 was not affected by the Lys417Glu/Asn/Thr mutation, which is a prominent escape site found in VoCs like B.1.351 (Vanshylla et al., 2022).

Considering the current epitope into account, BA.2 and BA.4/5 differ by one residue Phe486Val situated in the far flexible loop of the RBM motif of RBD, which establishes an auxiliary π-cation bond with Ser60 near CDRL2 (Figure 3B top, and Figure 3B), yet BA.2 but not BA.5 is neutralized by R40-1C8. Our previous findings suggest that this loop in RBM is highly flexible and plays a crucial role in ACE2 recognition and binding (Shah et al., 2020). The potential role of Phe486Val mutation and its resistance to R40-1C8 neutralization was further validated by the huge difference in total BFE of BA.5 against other Omicron subvariants and RBD^Wu01^ as well (Figure 3C; Supplementary Figure S1B). The rise in total BFE (in other words destabilization of the R40-1C8-RBD^BA.5^ complex) could be attributed to the loss in ELE potential. This was further validated by MMGBSA-based BFE calculation where the ELE potential raised from −169.33 kcal/mol (RBD^Wu01^-R40-1C8) to −74.77 kcal/mol (RBD^BA.5^-R40-1C8) (Supplementary Figure S2). We went on to investigate the interface alteration induced by BQ.1.1 and XBB.1.5 variants and calculated the loss in binding affinity using MMGBSA method. Like BA.5 where the total binding affinity dropped by ∼21.0 kcal/mol, the affinity of both BQ.1.1 and XBB.1.5 towards R40-1C8 dropped by 28 kcal/mol (Supplementary Figure S2). Surprisingly, the electrostatic potentials in both cases dropped by ∼170 kcal/mol.

To look further, we extracted 1,000 frames from the 50 ns MDS trajectory and considered the relative positioning of Phe486 in BA.1 and Val486. The heavy chain of antibody remained intact in BA.1- R40-1C8 complex but dissociated in BA.5-R40-1C8 complex (Supplementary Video S2), suggesting that in addition to electrostatic contacts, van der Waals forces also play a crucial role in Ag-Ab stability. Upon hydrogen bond network investigation, there was a steep decline in overall hydrogen bonds between BA.5-R40-1C8 complexes, which was slightly restored in the last quarter of the MDS course, perhaps due to new bonds formed between the VL chain and RBD (Figure 3D). Altogether, the epitope mapping and BFE suggest that Omicron subvariants like BA.5, BQ.1/.1, and XBB.1.5 containing Phe486-to-Val/Pro mutations are highly likely to escape the R40-1C8 neutralization. Further mutations at the R40-1C8-RBD interface were created on RBD to estimate/speculate the escape of future variants (Supplementary Table S4).

### R200-1F9 and R207-2F11 neutralize all Omicron variants

**FIGURE 4:**
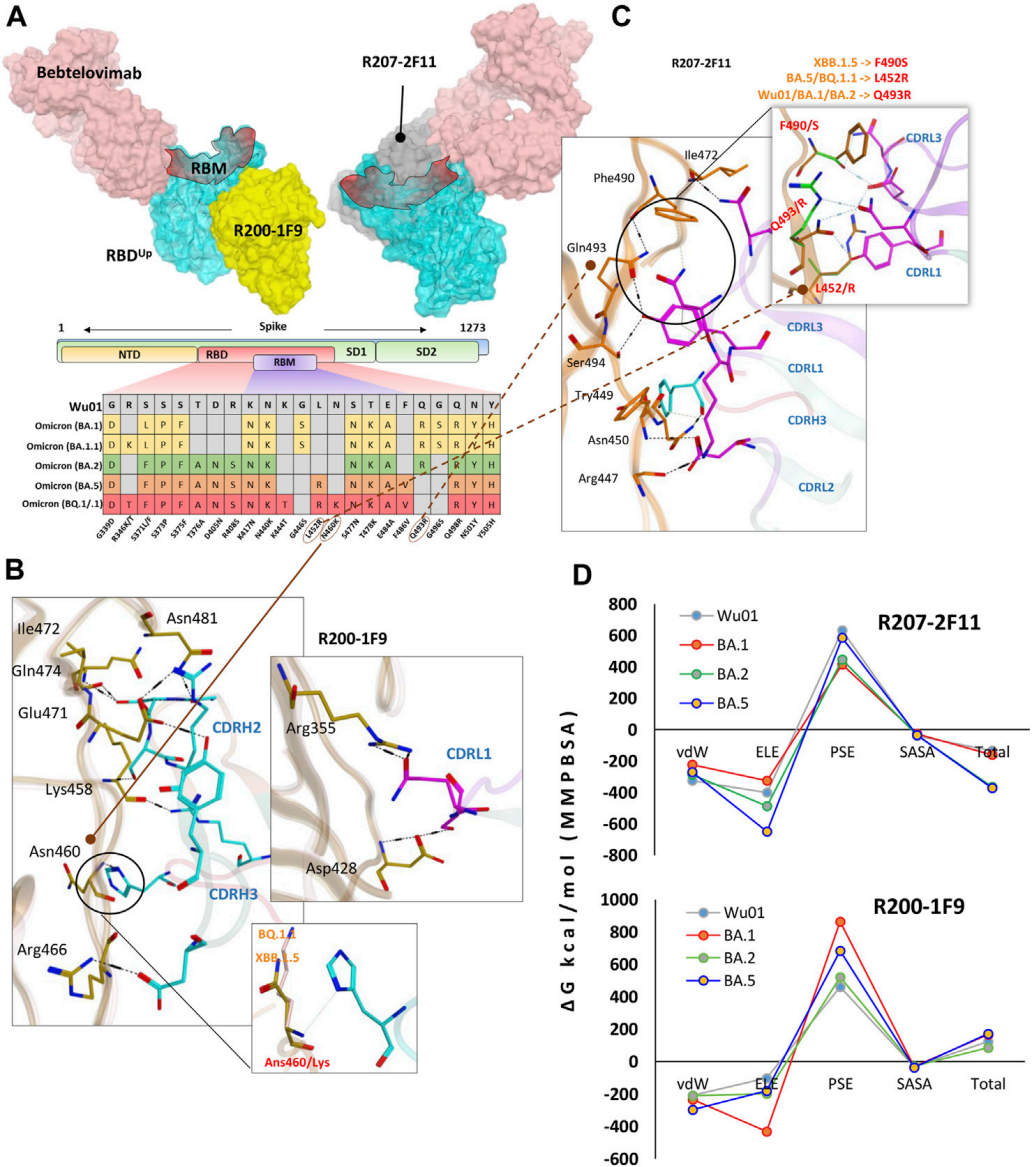
R200-1F9 and R207-2F11 neutralizes all Omicron variants. **(A)** The surface maps (colored according to chains) shows that both R200-1F9 and R207-2F11 do not compete with Bebtelovimab. Mutations in the RBD region of Spike protein of SARS-CoV-2 Omicron variants and their effect on the interface contacts with respect to R200-1F9 and R207-2F11. **(B)** Interface residues of the R200-1F9 with respect to Omicron variants. **(C)** Interface residues of the R207-2F11 with respect to Omicron variants. **(D)** Changes in the binding free energy (calculated through MMPBSA) of both R200-1F9 and R207-2F11 bound to the SARS-CoV-2 variants BA.1-B.5.

As discussed above, R200-1F9 can potentially bind to the RBD^Up^ only without competing with ACE2, whereas R207-2F11, a class II RBD nAb can potentially bind a conserved epitope on RBD in both ‘up’ and ‘down’ conformation. A co-model suggests that both R200-1F9 and R207-2F11 could be used as cocktail therapy with Bebtelovimab as they do not compete on RBD (Figure 4A). Since both R207-2F11 and R200-1F9 do not interfere with ACE2 directly, they possibly neutralize the virus by restricting the flexibility of RBM as most of their epitope residues are located adjacent to this motif (Supplementary Figure S3). Interface analyses indicate that the R200-1F9-RBD complex is stabilized by two salt bridges between Arg466 and Glu471 and CDHR2, which remains unchanged in all Omicron subvariants (Supplementary Table S5). However, His104 in CDRH3 creates a hydrogen bond with the backbone nitrogen of Asn460 which has been reported to be mutated into Lys460 in BQ.1.1 and XBB.1.5 variants. To explore whether Asn460/Lys affect this interaction, we constructed the RBD models of BQ.1.1 and XBB.1.5 and observed that the backbone nitrogen retains this bond in both cases (Figure 4B, bottom and Supplementary Table S5).

Unlike R200-1F9 where two strong electrostatic contacts were involved, most of the interface contacts were hydrogen bonds and other auxiliary forces in the R207-2F11-RBD complex, contributed by all three CDRs in light chains and a single hydrogen bond by CDRH3 (Figure 4C). R207-2F11 retained its neutralization effect against all previously reported Omicron subvariants (Gruell et al., 2022b); this was also supported by our BFE calculation where BA.2 and BA.5 substantially enhanced their binding affinity compared to Wu01 strain (Figure 4D). One of the most prominent mutations we observed was Leu452Arg in BA.5 and BQ.1.1 which established a salt bridge with the backbone oxygen of CDRL3 Asp92 (Supplementary Table S6). In addition, the loss of cation-π interaction between CDRL1 Asn30 and RBD Phe490 was substituted by a considerably strong Ser^490^-Asp^92^ hydrogen bond in RBD^XBB.1.5^ (Figure 4C). As BA.5 and BQ.1.1 variants contain Leu452Arg and other substitutions that enhance the antibody affinity, we suggest the R207-2F11 broad-nAb potentially retains its neutralization against XBB.1.5 (Leu452 is not mutated) and BQ.1.1 variants; however, this notion may require further experimental validation.

Unlike R40-1C8, which showed a significant decrease in binding affinity for all Omicron variants, and R401G8, which lost affinity only against BA.5, both R200-1F9 and R207-2F11 were found to have a similar or improved binding affinity for all Omicron variants of RBD compared to the RBD^Wu01^ (Figure 4D). This data was further validated by MMGBSA-based BFE calculation, where both XBB.1.5 and BQ.1.1 substantially enhanced their overall binding affinity against R207-2F11 and R200-1F9 (Supplementary Figure S4). Previous studies have shown that most broad-spectrum nAbs remain effective against single amino acid variants in the virus, but multiple mutations in the same epitope can reduce their efficacy. In the cases of the new BQ.1.1 and XBB.1.5 variants, many of the mutations occur outside the binding epitopes of these mAbs. The preservations of BFEs and the nature of auxiliary contacts made by single amino acid mutations within the epitopes suggest that these broad-spectrum nAbs may still be effective against the new Omicron variants of BQ.1.1 and XBB.1.5. However, to predict what other mutations would affect the neutralization ability of these mAbs, we created mutations on RBD at the R200-1F9/R207-2F11-RBD interface to estimate/speculate the escape of future variants (Supplementary Table S4).

### The escape of Omicron BQ.1.1 and XBB.1.5 from Bebtelovimab

**FIGURE 5:**
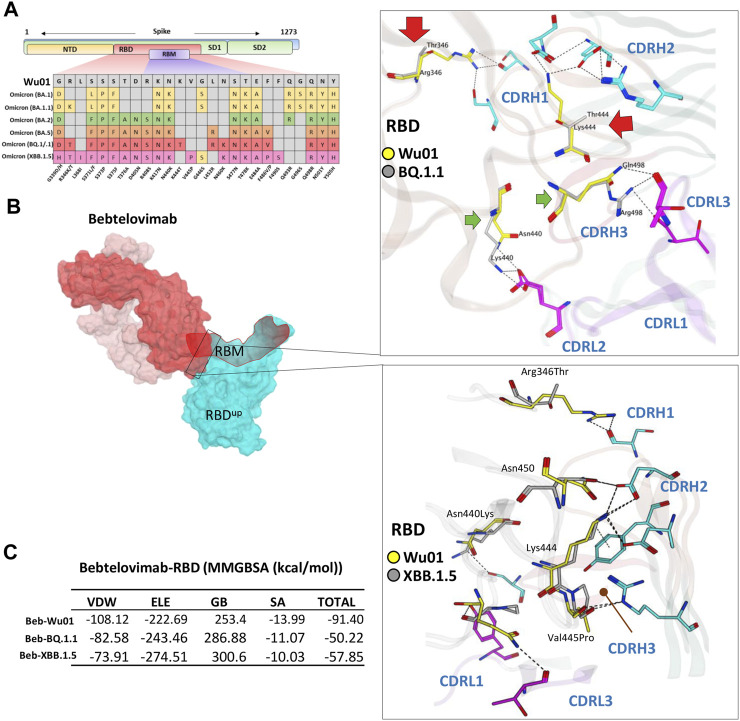
The escape of Omicron BQ.1.1 and XBB.1.5 from Bebtelovimab. **(A)** Mutations in the RBD region of Spike protein of SARS-CoV-2 Omicron variants and their effect on the interface contacts with respect to Bebtelovimab. **(B)** Mutations in both BQ.1.1 and XBB.1.5 abolish the Bebtelovimab interface. **(C)** Changes in the binding free energy (BFE, calculated through MMGBSA). Bebtelovimab loses its binding affinity against both new variants.

Among all available mAbs, Bebtelovimab stands out to be the best and has shown remarkable activity against all SARS-CoV-2 variants that have been reported until recently, including BA.4/5 (Imai et al., 2023). However, the emergence of BQ.1.1 a subvariant of the BA.5, and subvariants XBB and XBB.1.5 of the BA.2 have shown some extraordinary spread across multiple countries including the United States and India (Tamura et al., 2022). Their ancestors have already shown less sensitivity to a broad range of FDA-approved antibodies (Shah and Woo, 2021) and additional mutations in the RBD have put the neutralization potential of active nAbs like Bebtelovimab at further risk (Figure 5A).

To address this issue and predict their potential neutralization escape, we built the RBD models of both BQ.1.1 and XBB.1.5 bound to Bebtelovimab and calculated their BFE and interface changes. The potential of Bebtelovimab to retain its neutralization against a broad-range SARS-CoV-2 variants till now is attributed to its relatively conserved epitope on RBD as well as the utilization of all six CDRs in antigen binding (Figure 5B; Table 2). However, the emergence of BQ.1.1 with multiple mutations within its epitope could render this antibody ineffective. Four substitutions including Arg346Thr, Lys444Thr which abolished the strong electrostatic bonds, and Gln498Arg and Asn440Lys which established relatively stronger contacts with Bebtelovimab are of particular concern. Arg346Thr, Lys444Thr abolishes a network of salt bridges and hydrogen bonds with the CDRH1 and CDRH2 (Figure 5B). Similarly, XBB.1.5 which emerged with multiple new substitutions within the Bebtelovimab epitope, seems to weaken the Ag-Ab interface. Since XBB.1.5 is a descendent of BA.2, the Salt bridges established by Lys444 in RBD^Wu01^ with CDRH2 remained intact; however, there are two more substitutions Val445Pro and Gly446Ser in the same loop containing Lys444, which may restrict the loop flexibility, resulting in the reduced CDR-fitting. This model, RBD^XBB.1.5^-Bebtelovimab, was subjected to a 100 ns MDS to see the interface changes. As suggested, the interface contacts dropped by half (total of 7 hydrogen bonds) as compared to the RBD^Wu01^ (13 hydrogen bonds) (Table 2). Surprisingly, the MMGBSA-based BFE dropped by 45% in RBD^BQ.1.1^-Bebtelovimab, as compared to RBD^Wu01^, suggesting that Bebtelovimab is escaped by this variant. In the case of RBD^XBB.1.5^ the total BFE dropped to ∼36.26% and that of van der Waals energy dropped by ∼30% (Figure 5C). During the course of this study, multiple preliminary reports confirmed that both BQ.1.1 and XBB.1.5 escaped many anti-COVID-19 antibodies that are either approved by FDA, e.g., Bebtelovimab, or currently undergoing clinical investigations (Entzminger et al., 2023; Imai et al., 2023). Here we noticed that, although mutations such as Gln498Arg and Asn440Lys which rather strengthen the Bebtelovimab interface with these variants are surpassed by other mutations like Arg346Thr, Lys444Thr, Val445Pro, and Gly446Ser in one way or another, ultimately abrogating their interfaces, as suggested by the single amino acid energy contribution (Supplementary Figure S4). This further strengthens the concept that single mutations in a relatively conserved epitope could be endured by a broad-nAb; however, multiple mutations induce considerable conformational alterations in the epitopes that are beyond the CDRs fitting capacity.

**TABLE 2:** The change in energy contribution per residue at the RBD-Bebtelovimab interface and the effect of mutations in BQ.1.1 and XBB.1.5 variants.

Bebtelovimab-RBD (Wu01)						Bebtelovimab-RBD (BQ.1.1)						Bebtelovimab-RBD (XBB1.5)					
Type	mAb	RBD	Energy	Dist	BB	Type	mAb	RBD	Energy	Dist	BB	Type	mAb	RBD	Energy	Dist	BB
H	Ser30	Arg346	−8.5	2.87	b-	IH	Glu53	Lys440	−35.07	2.71	—-	H	Ser103	Lys440	−0.7	3.28	—-
H	Ser32	Arg346	−4.1	2.83	—-	H	Arg60	Val445	−2.5	2.79	b	IH	Asp56	Lys444	−32.43	2.84	—-
H	Glu53	Asn440	−5.5	2.85	—-	H	Thr96	Gly446	−0.5	3.45	bb	IH	Asp58	Lys444	−19.7	2.73	—-
IH	Asp56	Lys444	−27.71	2.96	—-	H	Arg60	Gly447	−8.6	2.77	b	H	Arg60	Pro445	−6.7	2.61	b
IH	Asp58	Lys444	−22.05	2.77	—-	H	Asp56	Asn450	−8.2	2.74	—-	H	Arg60	Gly447	−6.1	2.85	b
H	Arg60	Val445	−1.6	2.84	b	H	Thr95	Arg498	−1.8	2.76	b-	H	Asp56	Asn450	−8.6	2.81	—-
H	Thr96	Gly446	−0.8	3.25	bb	H	Thr96	Arg498	−1	2.97	b-	H	Tyr35	Pro499	−0.7	2.98	b
H	Arg60	Gly447	−8.4	2.75	b	H	Asp32	Thr500	−4.6	2.94	bb						
H	Asp56	Asn450	−7.2	2.7	—-	H	Asp32	Gly502	−0.6	3.43	b						
H	Thr96	Gln498	−4	2.81	b-												
H	Asp32	Thr500	−4.5	2.78	bb												
H	Gly31	Thr500	−0.5	3.33	bb												
H	Asp32	Gly502	−0.5	3.44	b												

mAb, monoclonal antibodies; RBD, receptor binding domain; Dist, bond length; BB, if backbone atoms are involved in bonding; Unit for energy is kcal/mol.

## Discussion

Neutralizing antibodies have become an essential form of treatment for individuals who have been diagnosed with severe COVID-19, particularly in cases where vaccination is not a viable option due to high-risk factors. The emergence of new variants has led to a decrease in the efficacy of mAbs in some cases, while in others, mAbs have displayed resistance. This is largely due to the different classifications of mAbs, which are based on the epitopes they recognize on the receptor binding domain of the virus. Some mAbs, such as Sotrovimab, have been observed to retain their neutralization capabilities against certain variants, such as Omicron BA.1, but show a reduced efficacy against others, such as BA.2, BA.4, BA.5, and BA.2.12.1 (Cox et al., 2023). Meanwhile, other variants, such as BQ.1.1, have been found to resist the neutralizing effects of mAbs like Sotrovimab, Bebtelovimab, and even combination therapies, such as Bamlanivimab-etesevimab or Evusheld (Imai et al., 2023). Additionally, the XBB.1.5 variant, which is considered a leading immune evasion variant, has been estimated to be the most transmissible variant yet (Callaway, 2023) and can evade all neutralizing antibodies (Imai et al., 2023).

The process of mAbs recognition and binding with antigens is highly specific, and even minor changes in the epitope-paratope interface can negatively impact this recognition and binding. With the appearance of new SARS-CoV-2 variants, some mAbs have lost their neutralization ability due to a single amino acid substitution in the epitope, such as the Lys417Asn and Leu452Arg substitutions. However, not all observed epitope mutations result in increased mAb evasion. For example, the R40-1C8 epitope was evaded by the BA.5 variant but not by the BA.1 and BA.2 variants. Currently, there is no FDA-approved antibody therapy that effectively neutralizes the latest Omicron variants BQ.1.1 and XBB.1.5. To overcome this challenge, it is possible to use the existing antibody repertoire against all SARS-CoV-2 variants by mapping their epitopes and escape sites.

The current strategies for engineering neutralizing therapeutic mAbs usually involve isolating antibodies from infected or vaccinated individuals or immunized humanized mice (Morales-Nunez et al., 2021; Taylor et al., 2021). However, with the rapidly evolving SARS-CoV-2 virus, this process has become increasingly challenging, especially where the lead discovery phase must be repeated each time a new variant arises. Due to the potential of dangerous reinfection associated with new variants of SARS-CoV-2 (Bowe et al., 2022; Brown et al., 2022), there is an urgent need to develop broadly active mAbs with both prophylactic and therapeutic potential for high-risk patients (Sullivan et al., 2022; Arora et al., 2023). Computational methods such as antibody-antigen docking, epitope mapping, molecular dynamics simulation, and binding free energies calculations have been widely used to screen and design antibodies. These methods can help to identify potential antibody candidates, optimize their binding affinity, and understand the underlying mechanisms of antibody-antigen interactions. These computational methods have been demonstrated to be effective in identifying and designing antibodies with high affinity and specificity. For example, computational methods have been used to design antibodies against the Ebola virus that have shown high efficacy in animal models (West et al., 2019). In addition, computational methods have been used to optimize the binding affinity of existing antibodies, such as those against HIV and influenza (Bowe et al., 2022).

In this study, we used an approach that involves shape complementarity of epitope and paratope residues flexibly, supported by molecular dynamics simulations to confirm stability and resistance to conformational changes. The results suggest that even though BQ.1.1 and XBB.1.5 have multiple new mutations, they are located outside the epitopes of the R200-1F9 and R207-2F11 nAbs. Additionally, the auxiliary contacts made by single amino acid mutations within the epitopes of these mAbs suggest their resilience against both variants. The previously confirmed *in vitro* efficacy of these antibodies further supports the authenticity of the protocol used here (Gruell et al., 2022b; Vanshylla et al., 2022). While computational methods have proven to be valuable tools in antibody design, they also have limitations, such as the accuracy of the models and the need for experimental validation (Lippow et al., 2007). Additionally, there is a possibility of errors in the predictions made by the softwares used, as the accuracy of these predictions is influenced by the quality and completeness of the protein structures and the varying background algorithms. Furthermore, such computational approaches may not take into account the complex interplay between various components of the immune system and the virus, which may impact the efficacy of the identified mAbs *in vivo*. Despite these limitations, machine learning and deep learning techniques can be used to improve the accuracy and efficiency of epitope mapping and antibody design (Akbar et al., 2021; Shan et al., 2022).

In conclusion, our study suggests that there is potential to redirect existing mAbs against new SARS-CoV-2 variants by mapping their epitopes and escape sites. The approach used in this study, which involves shape complementarity and simulations to confirm stability and resistance to conformational changes, has proven to be effective in identifying mAbs with resilience against both BQ.1.1 and XBB.1.5 variants. Further research is necessary to validate these findings and explore the potential of this approach for the development of broadly active mAbs for the treatment and prevention of COVID-19.

## Materials and methods

### Structures modeling of the Omicron subvariants and monoclonal antibodies (mAbs)

Among the 126 cross-neutralizing mAbs, (Kumari et al., 2014) mapped the epitopes of R40-1G8 on the SARS-CoV-2 (Wu01) Spike and resolved their co-crystal structure (PDB ID: 7SC1) (Vanshylla et al., 2022). R40-1G8 was identified as a broadly neutralizing antibody, which was effective against B.1.1.7, B.1.351, B.1.429, B.1.617, and B.1.617.2, as well as 19 prominent potential escape sites in the RBD (Vanshylla et al., 2022). However, their recent study demonstrates that this antibody was less or not effective against the Omicron and its subvariants (Gruell et al., 2022b). To understand the underlying mechanism of this escape, we used the R40-1G8-RBD complex as starting structure and constructed a 3D protein model of R40-1G8-RBD of Omicron BA.1, BA.2, and BA.5 using MOE 2022 package, as described previously (Shah and Woo, 2021). For the construction of isolated RBD structures of Omicron’s subvariants, the RBD^BA.1^ (PDB ID: 7WBP) crystal structure was used template. Mutations in the Omicron subvariants were made according to the GSAID reported list as displayed in Supplementary Figure S1A. The structure of an ultra-potent mAb Bebtelovimab bound to Spike protein was retrieved from RSCB PDB (ID: 7MM0) to demonstrate the escape of BQ.1.1 Omicron variant.

While many Omicron subvariants escaped the mAbs isolated by Florian Klein’s group, some retained their broad-spectrum neutralization against the BA.1, BA.2 and BA.5 variants of Omicron (Gruell et al., 2022b). We selected three mAbs from this study including R40-1C8, R207-2F11, and R200-1F9, which were effective against at least four Omicron subvariants and Wu01 strains, for their epitope mapping against the new variants. The amino acid sequences of all mAbs (variable fragment (FV) regions) including R40-1C8, R207-2F11, and R200-1F9, were retrieved from the coronavirus antibody database (CoV-AbDab) (Raybould et al., 2021) and their CDRs were numbered and annotated in MOE 2022, according to the IMGT system as previously described (Lefranc et al., 2005). The 3D structural model of all mAbs was constructed using default parameters in the MOE antibody modeler package. For each antibody model, the best templates for Framework and CDRs were selected from the built-in antibody database following best scoring, amino acids similarities, and % identity criteria. All constructed models were neutralized in a cubical solvent environment and energy was minimized following the antibody modeling suggested protocol in MOE. Amino acid sequences of the antibodies investigated in this study are available in Supplementary Data. For Bebtelovimab and related analysis, the crystal structure of mAb-bound RBD^Wu01^ was retrieved from RSCB PDB (PDB ID: 7MMO) (Westendorf et al., 2022).

### Antigen-antibodies docking and epitopes mapping

To map the epitopes of broadly neutralizing mAbs R40-1C8, R207-2F11, and R200-1F9 on SARS-CoV-2 Spike, we used three different protocols to authenticate the outcomes of our analyses. First, a widely implemented DiscoTope (conformational B cell epitope prediction package in IEDB resource) server was used to predict and annotate the potential spatial epitopes on SARS-CoV-2 RBD (Kringelum et al., 2012). Second, the epitope residues suggested by DiscoTope were validated through a linear epitope predictor, Bepipred (IEDB) (Clifford et al., 2022). Finally, these epitopes were confirmed by corresponding to that reported in actual RBD-mAbs structures of R40-1G8-RBD (ID: 7SC1) and Bebtelovimab-RBD (ID: 7MM0).

To predict the epitopes of R40-1C8, R207-2F11, and R200-1F9, robust antibody docking was used as described previously (Shah et al., 2020). For each mAb-RBD complex, 50 conformations were generated by Ag-Ab docking package in Molecular Operating Environment (MOE 2022.02) (Chemical computing group, Montreal, CANADA), where the ligands site were restricted to CDRs and for Ag, RBD was considered as a whole. A protein-ligand interaction fingerprint (PLIF) was generated based on 50 conformations of each mAb which summarized the contribution of each amino acid at the Ag-Ab interface. Based on PLIF results, five epitopes, ranked according to the docking score (kcal/mol), were suggested for each antibody which was further investigated for conservancy in SARS-CoV-2 variants and immunogenicity. As the subvariants neutralization of these mAbs have already been confirmed *in vitro* (Gruell et al., 2022b; Vanshylla et al., 2022), mAbs-RBD conformers that were in line with experimental data, bound to conserved epitopes, and establishing significant electrostatic and van der Wall contacts were selected, manually investigated, and further subjected to extensive molecular dynamics simulations for conformational stability and binding affinity estimation.

### Molecular dynamics simulations

Protein models were simulated in a cubic box containing the TIP3P solvent model using GROMACS 2022 under the CHARM36 force field (Huang et al., 2017). Models were centered in the box and neutralized with Na and Cl ions, as well as an additional 0.1M concentration of NaCl. The systems were first energy minimized, then equilibrated under constant temperature (NVT) and constant pressure (NPT) conditions for 0.5 ns. To prevent systems’ breakage, proteins and solvents were separated and constraints were applied to protein atoms. During the NVT step, the temperature was coupled with the v-rescale (modified Berendsen) thermostat, while the unmodified Berendsen algorithm was used in the NPT step (Bussi et al., 2007). All systems were simulated for at least 50 ns without structural constraints, using the Particle Mesh Ewald algorithm to calculate long-range electrostatic interactions (Wang et al., 2010). After the simulation, artifacts were removed from the MD trajectories using the -PBC and -fit flags in the trjconv tool, along with various functions such as whole, nojump, and rot + trans. Similar molecular dynamics simulation parameters have also been used by other groups to estimate the conformational changes and binding affinities of macromolecules with their ligands (Zhao et al., 2020; Vargas et al., 2020).

### Binding affinity estimation using binding free energies calculation

To calculate binding energies, we used two methods: the endpoint binding free energy MMGBSA method using the HawkDock server (Feng et al., 2017) and the free energy perturbation method using MMPBSA implemented in GROMACS (versions 5.0 and earlier) (Kumari et al., 2014). The MMPBSA method is particularly well-suited for calculating the binding energies of various ligands to the same target. The newer versions of GROMACS are not compatible with MMPBSA, so we generated the topology files for each Ab-Ag complex using GROMACS version 5.0. We analyzed an optimized simulation trajectory containing 100 frames to calculate binding free energies, as described in a previous study (Shah et al., 2020). Alanine mutagenesis was performed on the DruScorePPI server as described previously (Kruger and Gohlke, 2010).

## Data Availability

The original contributions presented in the study are included in the article/Supplementary Material, further inquiries can be directed to the corresponding author.
