# Supplementary material for: Assessment of neutralization susceptibility of Omicron subvariants XBB.1.5 and BQ.1.1 against broad-spectrum neutralizing antibodies through epitopes mapping
Source: Front Mol Biosci. 2023 Sep 27;10:1236617. doi: 10.3389/fmolb.2023.1236617 (PMC10565033; doi:10.3389/fmolb.2023.1236617)
Supplement: Supplementary file 3 [file DataSheet1.docx]

# **Assessment of neutralization susceptibility of Omicron subvariants XBB.1.5 and BQ.1.1 against broad-spectrum neutralizing antibodies through epitopes mapping**

Masaud Shah^1^ and Hyun Goo Woo^1, 2,^*

^1^Department of Physiology, Ajou University School of Medicine, Suwon 16499, Republic of Korea. ^2^Department of Biomedical Science, Graduate School, Ajou University, Suwon 16499, Korea

**Supplementary data**

**Supplementary Figures**

**Figure S1**

**
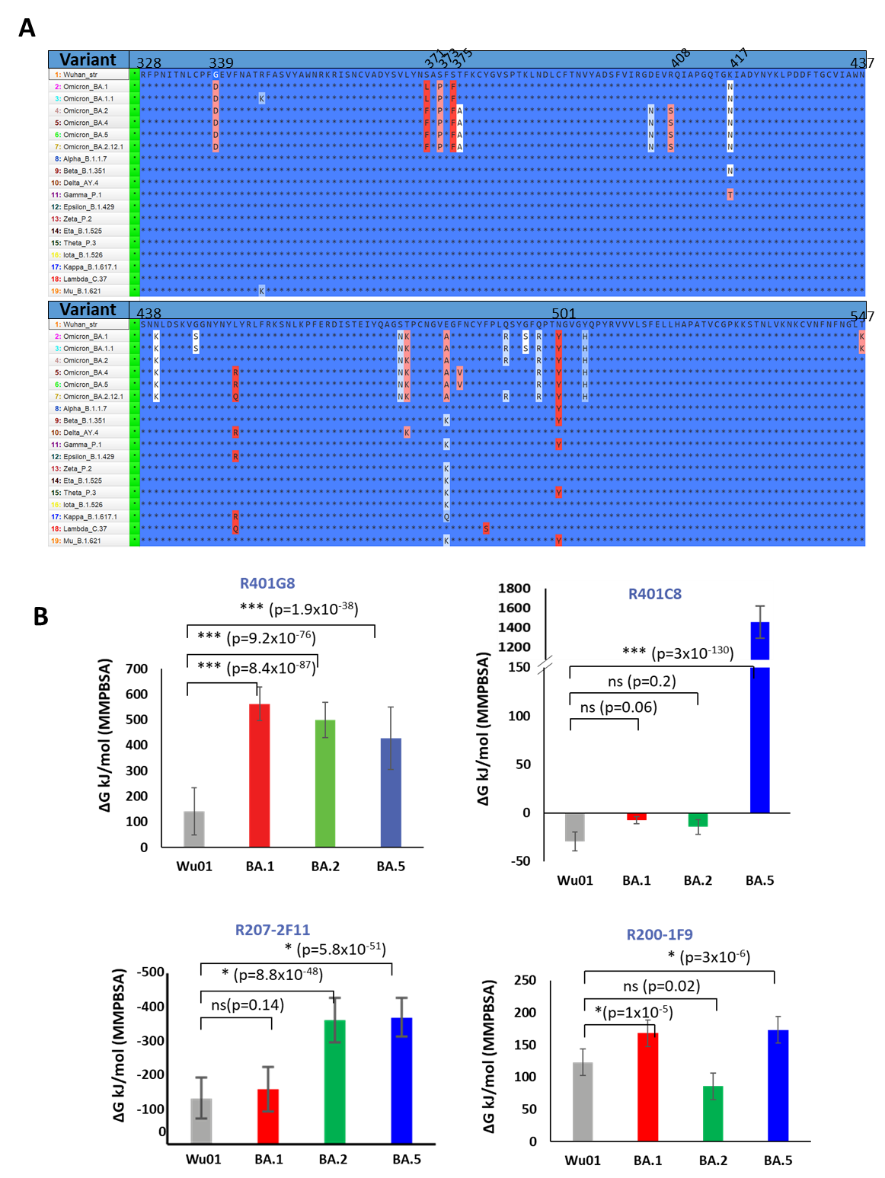
**

**Figure S1.** (A) Multiple sequence alignment and variations in the SARS-CoV-2 RBD region of Spike protein. (B) Changes in the total binding free energy of mAbs bound to the SARS-CoV-2 variants (RBD). The p value was calculated by two sample t-test. (p=0.005 was considered significant)

**Figure S2**


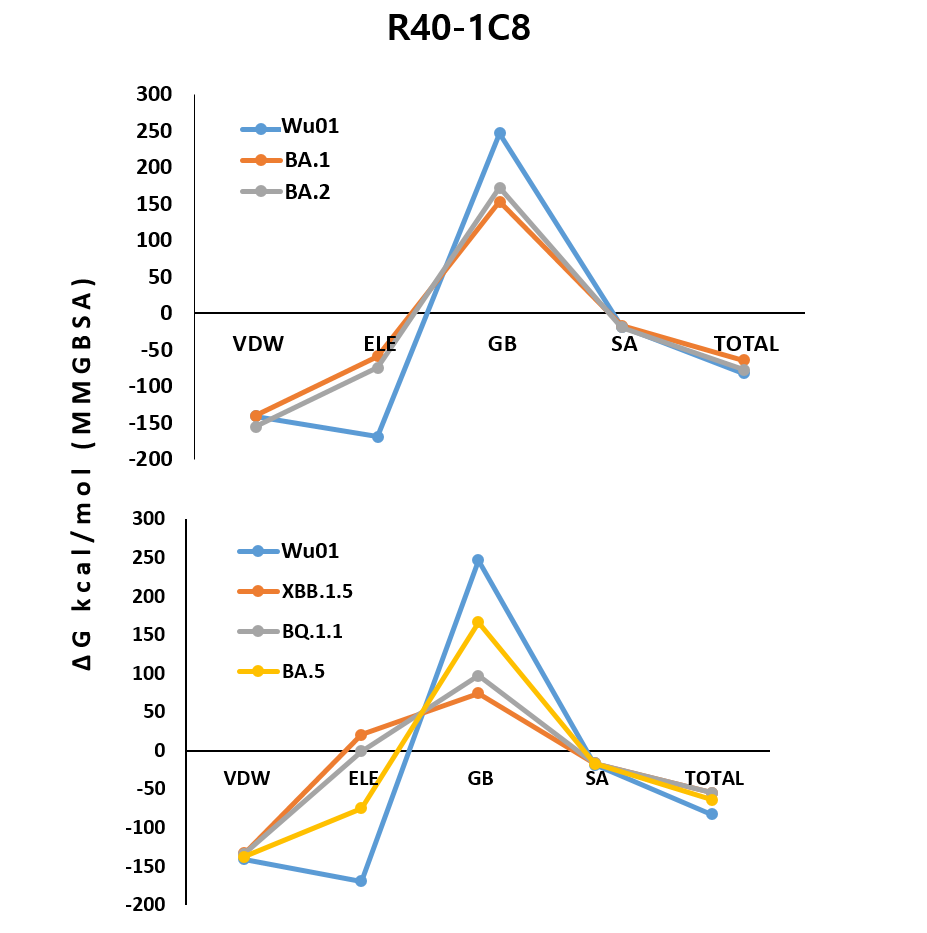


**Figure S2:** Changes in the binding free energy (through MMGBSA) of R40-1C8 bound to the SARS-CoV-2 Wu01 and Omicron variants.

**Figure S3**


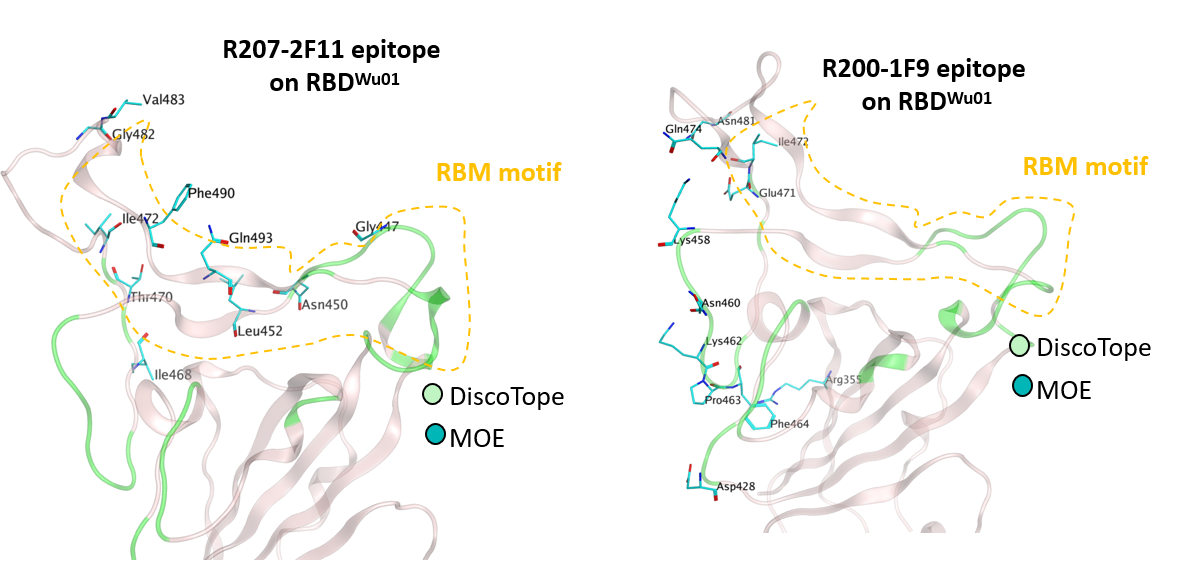


**Figure S3.** The epitope residues on RBD are predicted through DiscoTope (green color cartoon representation) and Molecular Operating Environment (MOE, Cyan color).

**Figure S4**


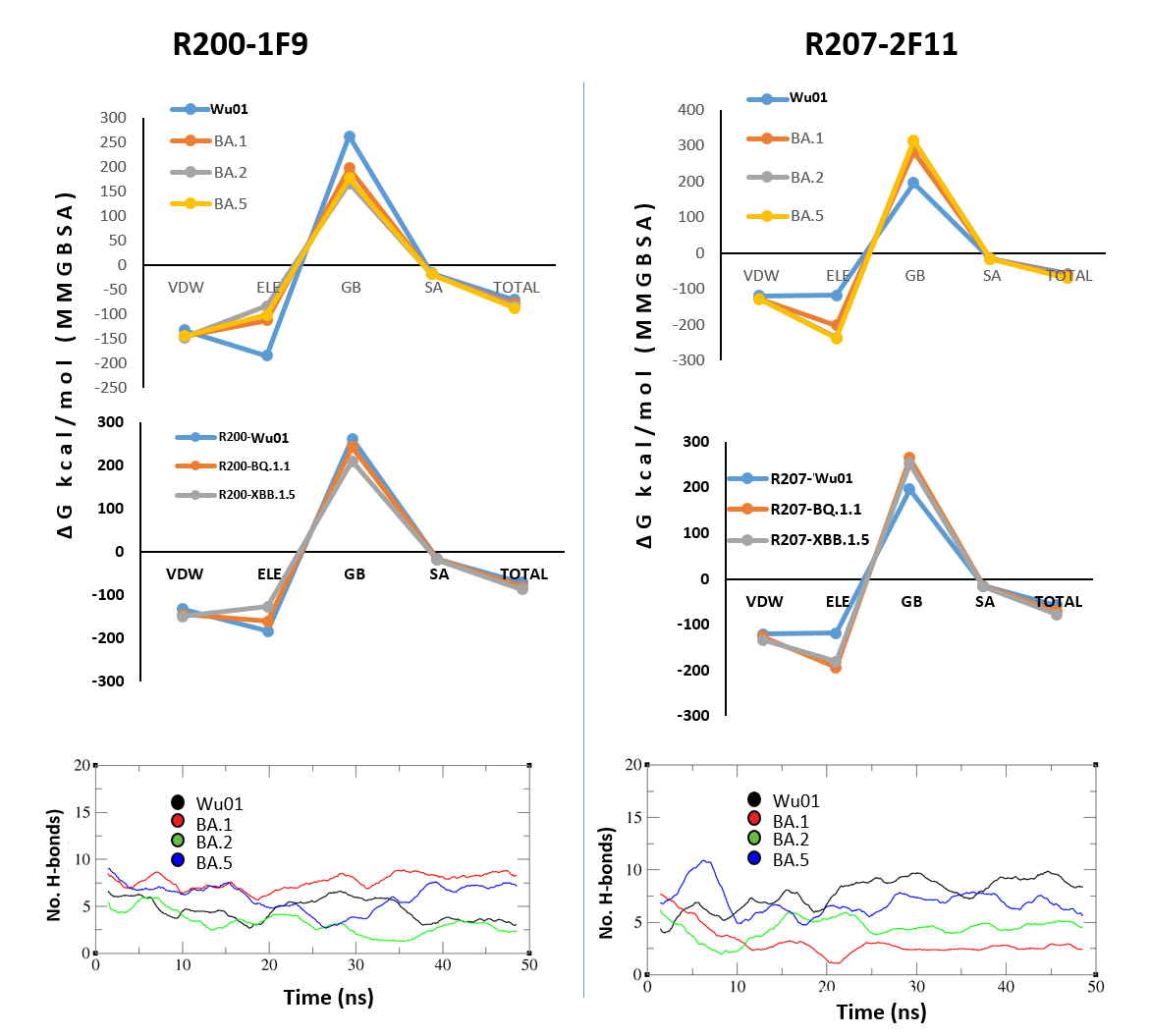


**Figure S4.** Changes in the binding free energy (MMGBSA) of R200-1F9 (*left top two panels*) bound to the SARS-CoV-2 variants. Bottom plot is showing changes in the number of Hydrogen bonds between R200-1F9 and Omicron variants. Changes in the binding free energy (MMGBSA) of R207-2F11 (*right top two panels*) bound to the SARS-CoV-2 variants. Bottom Plot showing changes in the number of Hydrogen bonds between R207-2F11 and Omicron variants.

**Figure S5**


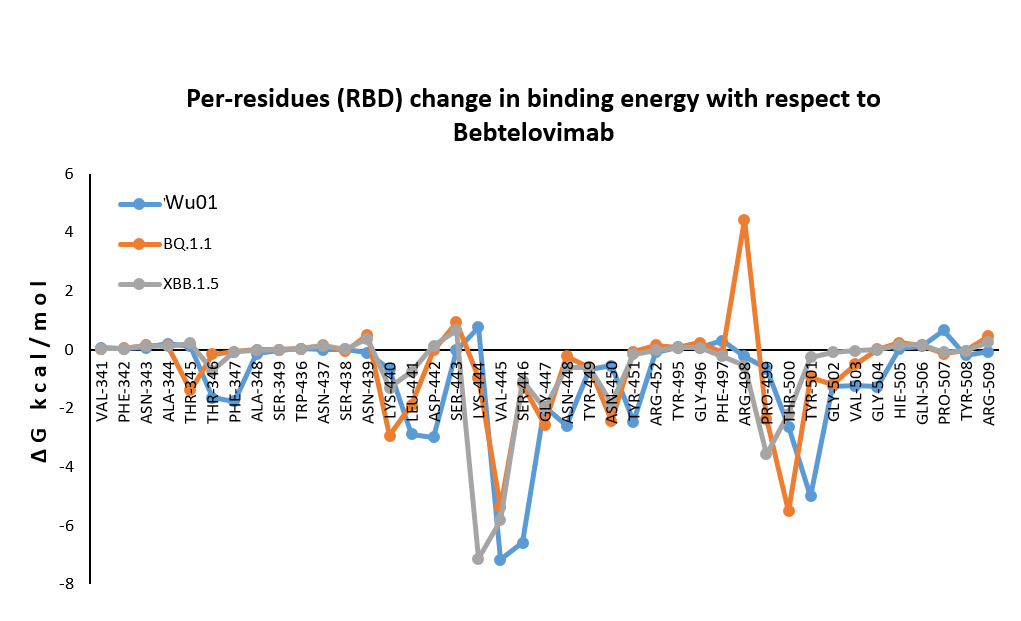


**Figure S5.** Per residues change in binding energy between Bebtelovimab and Omicron BQ.1.1 and XBB.1.5.

**Supplementary Tables**

**Table S1**

| **R40-1G8-RBD (Wu01)** | | | | | | **R40-1G8-RBD (BA1)** | | | | | | **R40-1G8-RBD (BA2)** | | | | | | **R40-1G8-RBD (BA5)** | | | | | |
| --- | --- | --- | --- | --- | --- | --- | --- | --- | --- | --- | --- | --- | --- | --- | --- | --- | --- | --- | --- | --- | --- | --- | --- |
| Type | mAb | RBD | Energy | Dist | BB | Type | mAb | RBD | Energy | Dist | BB | Type | mAb | RBD | Energy | Dist | BB | Type | mAb | RBD | Energy | Dist | BB |
| H | Ser93 | Arg403 | -2.8 | 3.05 | -- | H | Ser93 | Asp405 | -0.5 | 3.67 | b- | A | Tyr32 | Arg403 | -1.1 | 3.36 | -- | H | Asn92 | Arg403 | -3.5 | 2.87 | b- |
| H | Gln27 | Asp405 | -2.4 | 2.79 | -- | H | Asn92 | Glu406 | -2.6 | 2.85 | -- | H | Gln27 | Asn405 | -3.1 | 2.83 | -- | H | Ser93 | Asn405 | -1.9 | 2.94 | -- |
| IH | Asp94 | Arg408 | -27.7 | 2.73 | -- | IH | Asp94 | Arg408 | -12.93 | 2.7 | -- | H | Asp94 | Asn405 | -3.5 | 2.73 | -- | H | Asp94 | Gln409 | -4 | 2.78 | -- |
| H | Asp94 | Gln409 | -6.4 | 2.81 | -- | H | Asp94 | Gln409 | -6.1 | 2.79 | -- | H | Asn92 | Glu406 | -3.1 | 2.78 | -- | H | Ser56 | Asp420 | -3.1 | 2.67 | -- |
| H | Asn92 | **Lys417** | **-1.2** | **2.97** | **--** | H | Tyr52 | **Asn417** | **-2.4** | **2.69** | -- | H | Asp94 | Gln409 | -4.7 | 2.76 | -- | H | Gly55 | Tyr421 | -1.2 | 2.88 | b- |
| H | Asp94 | **Lys417** | **-7.8** | **2.8** | **b-** | H | Ser56 | Asp420 | -2.4 | 2.63 | -- | H | Tyr52 | Asn417 | -1.8 | 2.78 | -- | H | Asn92 | Tyr453 | -1.5 | 2.89 | -- |
| H | Ser56 | Asp420 | -0.5 | 2.59 | -- | H | Asn92 | Tyr453 | -1.3 | 2.66 | -- | H | Ser56 | Asp420 | -2.9 | 2.77 | -- | A | Tyr33 | Phe456 | -0.5 | 3.81 | -- |
| H | Tyr33 | Leu455 | -1.5 | 2.63 | b | H | Tyr33 | Leu455 | -3.3 | 2.64 | b | H | Asn92 | Tyr453 | -2 | 2.67 | -- | H | Ser31 | Lys458 | -7.4 | 2.77 | -- |
| H | Ser31 | Lys458 | -7.5 | 2.78 | -- | H | Ser30 | Lys458 | -0.5 | 2.79 | b- | H | Tyr33 | Leu455 | -3.4 | 2.67 | b | H | Ser31 | Tyr473 | -0.9 | 2.82 | b- |
| H | Ser56 | Asn460 | -2.4 | 2.84 | -- | H | Ser31 | Lys458 | -6.1 | 2.81 | -- | H | Ser31 | Lys458 | -5 | 2.79 | -- | H | Asn32 | Ala475 | -4.9 | 2.79 | b |
| H | Ser31 | Gln474 | -2.9 | 2.99 | b- | H | Ser31 | Tyr473 | -3.2 | 2.73 | b- | H | Gly55 | Asn460 | -2.1 | 2.9 | b- | H | Gly26 | Asn477 | -5.1 | 2.99 | b* |
| H | Arg97 | Phe486 | -8.3 | 2.86 | b | H | Ser31 | Gln474 | -3.1 | 2.65 | b | H | Ser31 | Tyr473 | -3.1 | 2.75 | b- | H | Tyr32 | Tyr495 | -2 | 2.71 | b |
| H | Gly26 | Asn487 | -3.3 | 2.69 | b- | H | Asn32 | Ala475 | -1.7 | 2.8 | b | A | Val2 | Phe486 | -0.6 | 4.5 | -- | H | Ser31 | Tyr501 | -1.7 | 2.81 | -- |
| H | Ser30 | Asn501 | -2.1 | 2.84 | -- | H | Gly26 | Asn477 | -2.5 | 3.07 | bb | H | Ser31 | Asn487 | -2.1 | 2.71 | -- | H | Gly28 | His505 | -4.1 | 2.82 | b- |
| H | Asn92 | Tyr505 | -2.1 | 2.7 | b- | H | Leu27 | Asn477 | -1.1 | 2.86 | b- | H | Asp98 | Tyr489 | -5 | 2.63 | -- |  |  |  |  |  |  |
|  |  |  |  |  |  | H | Tyr32 | Ser494 | -3.9 | 2.71 | b | A | Phe102 | Arg493 | -0.5 | 3.43 | -- |  |  |  |  |  |  |
|  |  |  |  |  |  |  |  |  |  |  |  | H | Tyr32 | Ser494 | -2.4 | 2.67 | b |  |  |  |  |  |  |
|  |  |  |  |  |  |  |  |  |  |  |  | H | Ser30 | Tyr495 | -0.5 | 3.12 | b |  |  |  |  |  |  |
|  |  |  |  |  |  |  |  |  |  |  |  | H | Ser31 | Arg498 | -3 | 3 | -- |  |  |  |  |  |  |
|  |  |  |  |  |  |  |  |  |  |  |  | H | Gly28 | Gly502 | -2.4 | 3.03 | bb |  |  |  |  |  |  |
| mAb, monoclonal antibodies; RBD, receptor binding domain; Dist, bond length; BB, if backbone atoms are involved in bonding; Unit for energy is kcal/mol. | | | | | | | | | | | | | | | | | | | | | | | |

**Table S1.** The change in energy contribution per residue at the RBD-R401G8 interface and the effect of mutations in BA.1-BA.5 variants.

**Table S2**

|  | VDW | ELE | GB | SA | TOTAL |
| --- | --- | --- | --- | --- | --- |
| Wu01 | -150.58 | -99.52 | 160.77 | -20.69 | -110.03 |
| BA.1 | -141.16 | 179.64 | -91.54 | -18.9 | -71.97 |
| BA.2 | -163.37 | 157.03 | -66.7 | -20.62 | -93.66 |
| BA.5 | -132.65 | 179.59 | -93.29 | -17.01 | -63.37 |

**Table S2. MMGBSA based binding free energy for RBD-R401G8 complexes of SARS-CoV-2 variants.**

**Table S3.**

| **R40-1C8-RBD (Wu01)** | | | | | | **R40-1C8-RBD (BA1)** | | | | | | **R40-1C8-RBD (BA2)** | | | | | | **R40-1C8-RBD (BA5)** | | | | | | **R40-1C8-RBD (BQ.1.1)** | | | | | | **R40-1C8-RBD (XBB.1.5)** | | | | | |
| --- | --- | --- | --- | --- | --- | --- | --- | --- | --- | --- | --- | --- | --- | --- | --- | --- | --- | --- | --- | --- | --- | --- | --- | --- | --- | --- | --- | --- | --- | --- | --- | --- | --- | --- | --- |
| Type | mAb | RBD | Energy | Dist | BB | Type | mAb | RBD | Energy | Dist | BB | Type | mAb | RBD | Energy | Dist | BB | Type | mAb | RBD | Energy | Dist | BB | Type | mAb | RBD | Energy | Dist | BB | Type | mAb | RBD | Energy | Dist | BB |
| H | Tyr102 | Glu406 | -4.9 | 2.6 | -- | H | Thr53 | Arg403 | -0.6 | 3.24 | -- | H | Thr53 | Arg403 | -2.6 | 2.89 | -- | H | Thr53 | Arg403 | -3.8 | 2.88 | -- | H | Thr53 | Arg403 | -3.2 | 2.97 | -- | H | Thr53 | Arg403 | -3.2 | 2.97 | -- |
| H | Tyr33 | Gly413 | -2.8 | 2.93 | b- | H | Tyr102 | Glu406 | -4.1 | 2.78 | -- | H | Asp101 | Asn405 | -1.8 | 2.76 | -- | H | Asp101 | Asn405 | -3.4 | 2.74 | -- | H | Asp101 | Asn405 | -2.5 | 2.75 | -- | H | Asp101 | Asn405 | -2.5 | 2.75 | -- |
| A | Tyr49 | Lys417 | -0.7 | 4.6 | -- | H | Leu99 | Arg408 | -2.5 | 3.19 | b- | H | Tyr102 | Glu406 | -1.2 | 2.54 | -- | A | Tyr102 | Asn405 | -0.7 | 3.6 | -- | A | Tyr102 | Asn405 | -0.7 | 3.86 | -- | A | Tyr102 | Asn405 | -0.7 | 3.86 | -- |
| IH | Asp105 | Lys417 | -25.01 | 2.7 | -- | H | Tyr33 | Gly413 | -3.4 | 2.75 | b- | H | Tyr33 | Gly413 | -2.6 | 2.66 | b- | H | Tyr102 | Glu406 | -4.3 | 2.62 | -- | H | Tyr102 | Glu406 | -4 | 2.61 | -- | H | Tyr102 | Glu406 | -4 | 2.61 | -- |
| IH | Arg97 | Asp420 | -15.25 | 3.07 | -- | H | Asp98 | Thr415 | -1.3 | 2.95 | b- | H | Arg97 | Thr415 | -2 | 2.98 | -- | H | Tyr33 | Gly413 | -3.1 | 2.77 | b- | H | Tyr33 | Gly413 | -3.2 | 2.7 | b- | H | Tyr33 | Gly413 | -3.2 | 2.7 | b- |
| H | Glu1 | Lys458 | -3 | 3.06 | b- | IH | Arg97 | Asp420 | -22.09 | 2.85 | -- | IH | Arg97 | Asp420 | -11.71 | 3.27 | -- | H | Tyr33 | Thr415 | -0.9 | 3 | b- | H | Tyr33 | Thr415 | -1 | 3.29 | b- | H | Tyr33 | Thr415 | -1 | 3.29 | b- |
| H | Asn32 | Asn460 | -6.7 | 2.86 | -- | H | Glu1 | Lys458 | -9.7 | 2.77 | b- | H | Glu1 | Lys458 | -9 | 2.91 | b- | H | Arg97 | Thr415 | -2.6 | 2.87 | -- | H | Arg97 | Thr415 | -3 | 2.9 | -- | H | Arg97 | Thr415 | -3 | 2.9 | -- |
| A | Ser60 | Phe486 | -0.8 | 4.64 | b- | H | Asn32 | Asn460 | -6.5 | 2.86 | -- | H | Asn32 | Asn460 | -6.6 | 2.86 | -- | IH | Arg97 | Asp420 | -11.66 | 2.7 | -- | IH | Arg97 | Asp420 | -12.44 | 2.79 | -- | IH | Arg97 | Asp420 | -12.44 | 2.79 | -- |
| H | Val58 | Tyr489 | -2.1 | 2.79 | b- | A | Ser60 | Phe486 | -0.7 | 3.94 | b- | A | Ser60 | Phe486 | -1.7 | 4.37 | b- | H | Glu1 | Lys458 | -8.6 | 2.79 | b- | H | Glu1 | Lys458 | -5.8 | 3 | b- | H | Glu1 | Lys458 | -5.8 | 3 | b- |
| H | Thr53 | Tyr505 | -2.3 | 2.65 | -- | H | Val58 | Tyr489 | -4 | 2.7 | b- | H | Val58 | Tyr489 | -4.5 | 2.69 | b- | H | Asn32 | Asn460 | -6.1 | 2.92 | -- | H | Val58 | Tyr489 | -4.1 | 2.66 | b- | H | Val58 | Tyr489 | -4.1 | 2.66 | b- |
|  |  |  |  |  |  | H | Gly50 | His505 | -1.8 | 2.73 | b- | H | Asp101 | His505 | -10 | 2.91 | -- | H | Val58 | Tyr489 | -3.5 | 2.67 | b- | H | Asp101 | His505 | -8.2 | 2.82 | -- | H | Asp101 | His505 | -8.2 | 2.82 | -- |
|  |  |  |  |  |  |  |  |  |  |  |  |  |  |  |  |  |  | H | Asp101 | His505 | -8 | 2.82 | -- |  |  |  |  |  |  |  |  |  |  |  |  |
| mAb, monoclonal antibodies; RBD, receptor binding domain; Dist, bond length; BB, if backbone atoms are involved in bonding; Unit for energy is kcal/mol. | | | | | | | | | | | | | | | | | | | | | | | | | | | | | | | | | | | |

**Table S3.** The change in energy contribution per residue at the RBD-R401C8 interface and the effect of mutations in all Omicron variants.

**Table S4**

**Table S4.** Antibody escape prediction of the SARS-CoV-2 future variants by crating RBD mutants bound to R401C8, R200-1F9, or R207-1F11. Decrease in the daffinity and dstability (kcal/mol) together, indicates the resistance of mAbs against that mutation.

**Table S5**

| **R200-1F9-RBD (Wu01)** | | | | | | **R200-1F9-RBD (BA1)** | | | | | | **R200-1F9-RBD (BA2)** | | | | | | **R200-1F9-RBD (BA5)** | | | | | | **R200-1F9-RBD (BQ.1.1)** | | | | | | **R200-1F9-RBD (XBB.1.5)** | | | | | |
| --- | --- | --- | --- | --- | --- | --- | --- | --- | --- | --- | --- | --- | --- | --- | --- | --- | --- | --- | --- | --- | --- | --- | --- | --- | --- | --- | --- | --- | --- | --- | --- | --- | --- | --- | --- |
| Type | mAb | RBD | Energy | Dist | BB | Type | mAb | RBD | Energy | Dist | BB | Type | mAb | RBD | Energy | Dist | BB | Type | mAb | RBD | Energy | Dist | BB | Type | mAb | RBD | Energy | Dist | BB | Type | mAb | RBD | Energy | Dist | BB |
| H | Asn1 | Arg355 | -3.5 | 3.11 | *- | H | Gln27 | Arg355 | -9.1 | 2.79 | -- | H | Gln27 | Arg355 | -9.1 | 2.79 | -- | H | Gln27 | Arg355 | -9.1 | 2.79 | -- | H | Gln27 | Arg355 | -9.1 | 2.78 | -- | H | Asn1 | Phe464 | -1.3 | 2.83 | bb |
| H | Gln27 | Arg355 | -6.7 | 2.88 | -- | H | Ser28 | Asp428 | -4.4 | 2.82 | -- | H | Ser28 | Asp428 | -4.5 | 2.82 | -- | H | Ser28 | Asp428 | -4.4 | 2.82 | -- | H | Ser28 | Asp428 | -2.1 | 2.53 | -- | H | Gln27 | Arg355 | -9 | 2.82 | -- |
| H | Ser28 | Asp428 | -3.3 | 2.61 | -- | H | Ser57 | Lys458 | -2.4 | 2.96 | b | H | Ser57 | Lys458 | -2.4 | 2.96 | b | H | Ser57 | Lys458 | -2.4 | 2.96 | b | H | Ser30 | Asp428 | -4.6 | 2.63 | -- | H | Ser28 | Asp428 | -1.7 | 2.56 | -- |
| H | Arg101 | Lys458 | -6 | 2.87 | b- | H | Arg101 | Lys458 | -4.1 | 2.74 | b | H | Arg101 | Lys458 | -4.1 | 2.74 | b | H | Arg101 | Lys458 | -4.1 | 2.74 | b | H | Ser57 | Lys458 | -2.4 | 2.96 | b | H | Gly55 | Asn481 | -1.2 | 2.73 | b- |
| H | His104 | Asn460 | -3.6 | 3.01 | b- | H | His104 | Asn460 | -1 | 3.01 | b | H | His104 | Asn460 | -1 | 3.02 | b | H | His104 | Asn460 | -1 | 3.01 | b | H | Arg101 | Lys458 | -2.8 | 2.7 | b | H | Ser56 | Ile472 | -2.3 | 2.65 | b |
| H | Ser105 | Lys462 | -0.6 | 3.15 | -- | H | His104 | Lys462 | -6.4 | 2.94 | b- | H | His104 | Lys462 | -6.4 | 2.94 | b- | H | His104 | Lys462 | -6.4 | 2.94 | b- | H | His104 | Lys460 | -3.9 | 3.12 | b | H | Ser56 | Gln474 | -2.1 | 2.88 | b |
| H | Asn93 | Pro463 | -3.8 | 2.91 | b- | H | Ser105 | Lys462 | -0.6 | 3.25 | -- | H | Ser105 | Lys462 | -0.6 | 3.25 | -- | H | Ser105 | Lys462 | -0.6 | 3.25 | -- | H | His104 | Lys462 | -1.1 | 3.57 | b- | H | Ser56 | Asn481 | -2.4 | 3.14 | b* |
| H | Asn93 | Phe464 | -1 | 2.77 | b- | H | Asn1 | Phe464 | -1 | 2.79 | bb | H | Asn1 | Phe464 | -0.9 | 2.79 | bb | H | Asn1 | Phe464 | -1 | 2.79 | bb | IH | Asp62 | Arg466 | -19.5 | 2.8 | -- | H | Ser57 | Lys458 | -2 | 2.99 | b |
| IH | Arg58 | Glu471 | -30.8 | 2.97 | -- | IH | Asp62 | Arg466 | -14.1 | 3.13 | -- | IH | Asp62 | Arg466 | -14.1 | 3.13 | -- | IH | Asp62 | Arg466 | -14.2 | 3.12 | -- | H | Trp94 | Arg466 | -1.6 | 3.25 | bb | IH | Arg58 | Glu471 | -30.5 | 2.95 | -- |
| H | Tyr60 | Glu471 | -6 | 2.66 | -- | H | Trp94 | Arg466 | -2.8 | 3.16 | bb | H | Trp94 | Arg466 | -2.8 | 3.16 | bb | H | Trp94 | Arg466 | -2.8 | 3.16 | bb | IH | Arg58 | Glu471 | -33.2 | 2.87 | -- | H | Tyr60 | Glu471 | -6.2 | 2.69 | -- |
| H | Ser56 | Ile472 | -2.3 | 2.71 | b- | IH | Arg58 | Glu471 | -32.9 | 2.91 | -- | IH | Arg58 | Glu471 | -32.9 | 2.91 | -- | IH | Arg58 | Glu471 | -33 | 2.91 | -- | H | Tyr60 | Glu471 | -5.3 | 2.67 | -- | IH | Asp62 | Arg466 | -15.3 | 2.71 | -- |
| H | Ser56 | Gln474 | -1.3 | 3.15 | b- | H | Tyr60 | Glu471 | -4.7 | 2.67 | -- | H | Tyr60 | Glu471 | -4.7 | 2.67 | -- | H | Tyr60 | Glu471 | -4.7 | 2.67 | -- | H | Ser56 | Ile472 | -2.2 | 2.76 | b | H | Ser71 | Asn481 | -0.7 | 3.4 | b- |
| H | Gly55 | Asn481 | -1.4 | 3 | b- | H | Ser56 | Ile472 | -2.4 | 2.66 | b | H | Ser56 | Ile472 | -2.4 | 2.66 | b | H | Ser56 | Ile472 | -2.4 | 2.66 | b | H | Ser56 | Gln474 | -2.7 | 2.94 | b | H | Asn93 | Pro463 | -0.8 | 3.11 | b |
| H | Arg58 | Asn481 | -5.8 | 2.74 | -- | H | Ser56 | Gln474 | -2.2 | 2.9 | b | H | Ser56 | Gln474 | -2.2 | 2.91 | b | H | Ser56 | Gln474 | -2.2 | 2.91 | b | H | Gly55 | Asn481 | -2.9 | 2.76 | b- | H | Trp94 | Arg466 | -2.7 | 3.2 | bb |
|  |  |  |  |  |  | H | Gly55 | Asn481 | -1.7 | 2.8 | b- | H | Gly55 | Asn481 | -1.7 | 2.8 | b- | H | Gly55 | Asn481 | -1.7 | 2.8 | b- |  |  |  |  |  |  | H | Arg101 | Lys458 | -3.2 | 2.77 | b |
|  |  |  |  |  |  |  |  |  |  |  |  |  |  |  |  |  |  |  |  |  |  |  |  |  |  |  |  |  |  | H | His104 | Lys460 | -4.2 | 3.13 | b |
|  |  |  |  |  |  |  |  |  |  |  |  |  |  |  |  |  |  |  |  |  |  |  |  |  |  |  |  |  |  | H | His104 | Lys462 | -8.8 | 2.79 | b- |
| mAb, monoclonal antibodies; RBD, receptor binding domain; Dist, bond length; BB, if backbone atoms are involved in bonding; Unit for energy is kcal/mol. | | | | | | | | | | | | | | | | | | | | | | | | | | | | | | | | | | | |

**Table S5.** The change in energy contribution per residue at the RBD-R200-1F9 interface and the effect of mutations in all Omicron variants.

**Table S6**

| **R207-2F11-RBD (Wu01)** | | | | | | **R207-2F11-RBD (BA1)** | | | | | | **R207-2F11-RBD (BA2)** | | | | | | **R207-2F11-RBD (BA5)** | | | | | | **R207-2F11-RBD (BA.1.1)** | | | | | | **R207-2F11-RBD (XBB.1.5 )** | | | | | |
| --- | --- | --- | --- | --- | --- | --- | --- | --- | --- | --- | --- | --- | --- | --- | --- | --- | --- | --- | --- | --- | --- | --- | --- | --- | --- | --- | --- | --- | --- | --- | --- | --- | --- | --- | --- |
| Type | mAb | RBD | Energy | Dist | BB | Type | mAb | RBD | Energy | Dist | BB | Type | mAb | RBD | Energy | Dist | BB | Type | mAb | RBD | Energy | Dist | BB | Type | mAb | RBD | Energy | Dist | BB | Type | mAb | RBD | Energy | Dist | BB |
| A | Tyr101 | Ser349 | -0.6 | 3.78 | b | H | Tyr49 | Arg346 | -0.7 | 2.94 | -- | H | Tyr101 | Ala352 | -1.4 | 3.15 | b | H | Tyr101 | Ala352 | -1.4 | 3.16 | b | H | Tyr101 | Ala352 | -2 | 2.95 | b | H | Tyr101 | Ala352 | -0.8 | 3.33 | b |
| H | Lys31 | Gly447 | -9.4 | 2.73 | b | A | Tyr101 | Ser349 | -0.5 | 3.61 | b | H | Lys31 | Gly447 | -9.3 | 2.71 | b | H | Lys31 | Gly447 | -8.3 | 2.72 | b | H | Lys31 | Gly447 | -7.7 | 2.72 | b | H | Lys31 | Gly447 | -6.8 | 2.71 | b |
| H | Tyr49 | Asn450 | -3.7 | 2.97 | b- | H | Ser52 | Ser446 | -1.2 | 2.89 | -- | H | Asn53 | Asn448 | -0.5 | 2.96 | -- | H | Tyr49 | Asn450 | -4.4 | 2.91 | b- | H | Asn53 | Asn448 | -1 | 2.93 | -- | H | Asn53 | Gly447 | -1.5 | 3.06 | b |
| H | Asp50 | Asn450 | -1 | 3.09 | b | H | Lys31 | Gly447 | -10.9 | 2.73 | b | A | Lys31 | Tyr449 | -0.5 | 4.61 | -- | H | Tyr101 | Asn450 | -2.9 | 2.87 | b- | H | Asp50 | Tyr449 | -2.6 | 2.89 | b | H | Asp50 | Tyr449 | -1.3 | 3.11 | b |
| H | Tyr91 | Asn450 | -0.6 | 3.38 | b | H | Tyr49 | Asn450 | -3.7 | 2.93 | b- | H | Tyr49 | Asn450 | -4.3 | 2.9 | b- | H | Tyr91 | Arg452 | -4.1 | 2.67 | b- | H | Tyr49 | Asn450 | -1.8 | 3.26 | b- | H | Asp50 | Asn450 | -2.4 | 3.09 | b |
| H | Tyr101 | Asn450 | -4.3 | 2.83 | b- | H | Tyr91 | Asn450 | -1.3 | 3.24 | b | H | Tyr101 | Asn450 | -3 | 2.87 | b- | IH | Asp92 | Arg452 | -5.69 | 3.36 | *- | H | Tyr101 | Asn450 | -3.8 | 2.84 | b- | H | Tyr101 | Asn450 | -3.3 | 2.76 | b- |
| H | Tyr52 | Ile468 | -1.8 | 2.81 | b | H | Tyr101 | Asn450 | -5.1 | 2.85 | b- | H | Asn93 | Ile472 | -0.8 | 3.23 | b | H | Asn93 | Ile472 | -0.5 | 3.28 | b | H | Tyr91 | Arg452 | -3.4 | 2.7 | b- | A | Tyr32 | Leu452 | -0.6 | 3.99 | -- |
| H | Asn93 | Ile472 | -3.4 | 2.94 | b | H | Asn93 | Ile472 | -2.6 | 2.98 | b | H | Leu94 | Asn481 | -3.2 | 2.9 | b- | H | Leu94 | Asn481 | -3 | 2.92 | b- | H | Asp92 | Arg452 | -6.5 | 2.86 | b- | H | Tyr52 | Ile468 | -2.2 | 2.85 | b |
| H | Lys1 | Gly482 | -10.3 | 2.72 | bb | H | Lys1 | Asn481 | -2 | 3.02 | bb | H | Lys1 | Gly482 | -7.1 | 2.75 | bb | H | Lys1 | Gly482 | -6.2 | 2.78 | bb | H | Tyr52 | Ile468 | -2.5 | 2.86 | b | H | Asn93 | Ile472 | -1.5 | 2.8 | b |
| H | Asn30 | Gln493 | -2.1 | 2.92 | -- | H | Lys1 | Gly482 | -8.3 | 2.73 | bb | H | Asn30 | Arg493 | -5.8 | 2.83 | -- | H | Asn30 | Gln493 | -2 | 2.88 | -- | A | Asn93 | Tyr473 | -0.9 | 3.78 | -- | A | Asn93 | Tyr473 | -0.7 | 3.65 | -- |
| H | Tyr32 | Gln493 | -2.1 | 2.57 | -- | H | Asn30 | Arg493 | -5.6 | 2.76 | -- | H | Tyr32 | Ser494 | -1.1 | 2.78 | -- | H | Tyr32 | Gln493 | -1.7 | 2.59 | -- | H | Lys1 | Asn481 | -8.4 | 2.98 | bb | H | Lys1 | Gly482 | -10.4 | 2.93 | bb |
|  |  |  |  |  |  | H | Tyr32 | Ser494 | -1.1 | 2.79 | -- |  |  |  |  |  |  | H | Tyr32 | Ser494 | -1.1 | 2.73 | -- | H | Asn30 | Gln493 | -2.1 | 2.87 | -- | H | Gln27 | Val483 | -0.6 | 3.42 | b |
|  |  |  |  |  |  |  |  |  |  |  |  |  |  |  |  |  |  |  |  |  |  |  |  | H | Tyr32 | Gln493 | -1.3 | 2.62 | -- | H | Asp92 | Ser490 | -2.5 | 2.83 | -- |
|  |  |  |  |  |  |  |  |  |  |  |  |  |  |  |  |  |  |  |  |  |  |  |  | H | Tyr32 | Ser494 | -1.1 | 2.74 | -- | H | Asn93 | Ser490 | -2.5 | 2.98 | -- |
|  |  |  |  |  |  |  |  |  |  |  |  |  |  |  |  |  |  |  |  |  |  |  |  |  |  |  |  |  |  | H | Asn30 | Gln493 | -1.9 | 2.9 | -- |
|  |  |  |  |  |  |  |  |  |  |  |  |  |  |  |  |  |  |  |  |  |  |  |  |  |  |  |  |  |  | H | Tyr32 | Gln493 | -1.4 | 2.56 | -- |
|  |  |  |  |  |  |  |  |  |  |  |  |  |  |  |  |  |  |  |  |  |  |  |  |  |  |  |  |  |  | H | Tyr32 | Ser494 | -0.9 | 2.8 | -- |
| mAb, monoclonal antibodies; RBD, receptor binding domain; Dist, bond length; BB, if backbone atoms are involved in bonding; Unit for energy is kcal/mol. | | | | | | | | | | | | | | | | | | | | | | | | | | | | | | | | | | | |

**Table S6.** The change in energy contribution per residue at the RBD-R207-2F11 interface and the effect of mutations in all Omicron variants.
